# Neurologic effects of short-term treatment with a soluble epoxide hydrolase inhibitor after cardiac arrest in pediatric swine

**DOI:** 10.1186/s12868-020-00596-y

**Published:** 2020-10-31

**Authors:** Caitlin E. O’Brien, Polan T. Santos, Ewa Kulikowicz, Jennifer K. Lee, Raymond C. Koehler, Lee J. Martin

**Affiliations:** 1 Department of Anesthesiology and Critical Care Medicine, Johns Hopkins University School of Medicine, 1800 Orleans Street, Bloomberg Children’s Center Suite 6302, Baltimore, MD 21287 USA; 2 Department of Pathology, Johns Hopkins University School of Medicine, 600 N. Wolfe Street, Baltimore, MD 21287 USA; 3 Pathobiology Graduate Training Program, Johns Hopkins University School of Medicine, 1800 Orleans Street, Bloomberg Children’s Center Suite 6302, Baltimore, MD 21287 USA

**Keywords:** Neuroprotection, TPPU, Brain damage, Basal ganglia, Cell death, Piglet, Cardiac arrest

## Abstract

**Background:**

Cardiac arrest (CA) is the most common cause of acute neurologic insult in children. Many survivors have significant neurocognitive deficits at 1 year of recovery. Epoxyeicosatrienoic acids (EETs) are multifunctional endogenous lipid signaling molecules that are involved in brain pathobiology and may be therapeutically relevant. However, EETs are rapidly metabolized to less active dihydroxyeicosatrienoic acids by soluble epoxide hydrolase (sEH), limiting their bioavailability. We hypothesized that sEH inhibition would improve outcomes after CA in an infant swine model. Male piglets (3–4 kg, 2 weeks old) underwent hypoxic-asphyxic CA. After resuscitation, they were randomized to intravenous treatment with an sEH inhibitor (TPPU, 1 mg/kg; n = 8) or vehicle (10% poly(ethylene glycol); n = 9) administered at 30 min and 24 h after return of spontaneous circulation. Two sham-operated groups received either TPPU (n = 9) or vehicle (n = 8). Neurons were counted in hematoxylin- and eosin-stained sections from putamen and motor cortex in 4-day survivors.

**Results:**

Piglets in the CA + vehicle groups had fewer neurons than sham animals in both putamen and motor cortex. However, the number of neurons after CA did not differ between vehicle- and TPPU-treated groups in either anatomic area. Further, 20% of putamen neurons in the Sham + TPPU group had abnormal morphology, with cell body attrition and nuclear condensation. TPPU treatment also did not reduce neurologic deficits.

**Conclusion:**

Treatment with an sEH inhibitor at 30 min and 24 h after resuscitation from asphyxic CA does not protect neurons or improve acute neurologic outcomes in piglets.

## Background

Cardiac arrest is the most common cause of acute neurologic injury in children [1]. Although survival has improved over the past 2 decades [2], 40–60% of survivors have moderate-to-severe neurologic impairment at 1 year of recovery [3–5]. Furthermore, 25% of patients classified as a having a favorable neurologic outcome by traditional assessment have significant cognitive impairment on neuropsychological testing at 1 year recovery [6], suggesting that more subtle and pernicious impairments are common and possibly overlooked on routine evaluations. Currently, no targeted clinical therapies exist to protect the developing brain after cardiac arrest.

Epoxyeicosatrienoic acids (EETs) and other fatty acid epoxides are endogenous lipid signaling molecules widely produced by cytochrome P450 epoxygenases during arachidonic acid metabolism and possess anti-inflammatory properties [7]. The EETs are naturally upregulated in brain parenchyma during ischemia [8] and thought to play several key protective roles after brain injury, including attenuation of excitotoxicity [9], reduction of inflammation [10], regulation of cerebral blood flow [11, 12], and inhibition of glial [9] and neuronal [10, 13] apoptosis. However, EETs are rapidly metabolized by soluble epoxide hydrolase (sEH) to the less protective dihydroxyeicosatrienoic acids, limiting their ability to protect the brain after injury. Increasing EET levels by administration of an sEH inhibitor at the time of brain injury is one strategy to enhance neuroprotection.

Genetic deletion or pharmacologic inhibition of sEH protects against hypoxic injury in adult rodent models of ischemic cardiomyopathy [14], ischemic stroke [13, 15], and cardiac arrest [16, 17]. The mechanism of this protection is unknown, but increasing evidence suggests that attenuation of endoplasmic reticulum (ER) stress might be one possibility [18]. The effect of sEH inhibition after global hypoxic injury in developing brain has not been assessed. The objective of this study was to evaluate whether administration of the sEH inhibitor TPPU after CA protects neurons in basal ganglia and cerebral cortex and improves acute neurologic outcomes in a pediatric swine model.

## Results

### Baseline characteristics, hypoxic–asphyxic injury, and resuscitation variables

Forty-six piglets underwent randomization, of which 34 survived for 4 days and underwent neuropathologic evaluation (Table 1). With an 8-min asphyxia protocol, overall 4-day survival was 25% (2/8). Three piglets could not be resuscitated from CA, 1 could not be extubated, 1 died several hours after extubation, and 1 died from intractable seizures despite treatment with intravenous phenobarbital. The two surviving piglets were included in pathology outcomes. When the protocol was amended to 7 min of asphyxia, 4-day survival improved to 84% (32/38). Two piglets died during surgery prior to protocol initiation, 1 could not be resuscitated from CA, 2 could not be extubated due to hemodynamic instability and poor respiratory effort, and 1 died several minutes after TPPU administration, thought to be secondary to venous air embolism. Of the 12 animals that did not complete the 4-day recovery, 6 (50%) received at least one dose of study drug. The distribution of piglets that died after receiving at least 1 dose of vehicle or TPPU were: 2 Sham + vehicle, 1 Sham + TPPU, 1 CA + vehicle, and 2 CA + TPPU (*p *= 0.853).

**Table 1:** Allocation of pigs to the 4 experimental groups

Parameter	Total	Sham + Veh	Sham + TPPU	CA + Veh	CA + TPPU
Intent-to-treat	46	11	10	13	12
Did not complete 4 day protocol	12	3	1	4	4
Survived 4 day protocol	34	8	9	9	8
Reasons for death					
Could not be resuscitated	4	N/A	N/A	2	2
Could not be extubated	3	2	0	0	1
Poor perfusion after extubation	1	0	0	0	1
Refractory seizures	1	0	0	1	0
Severe hypotension prior to protocol initiation	2	1	0	1	0
Other	1	0	1^a^	0	0
Asphyxia duration in 4 d survivors					
7 min	15	N/A	N/A	8	7
8 min	2	N/A	N/A	1	1
Received ≥ 1 dose of vehicle/drug prior to death	6	2	1	1	2

CA, cardiac arrest; N/A, not applicable; Veh, vehicle

^a^Died suddenly after 2nd TPPU injection, likely secondary to venous air embolism

**Fig. 1:**
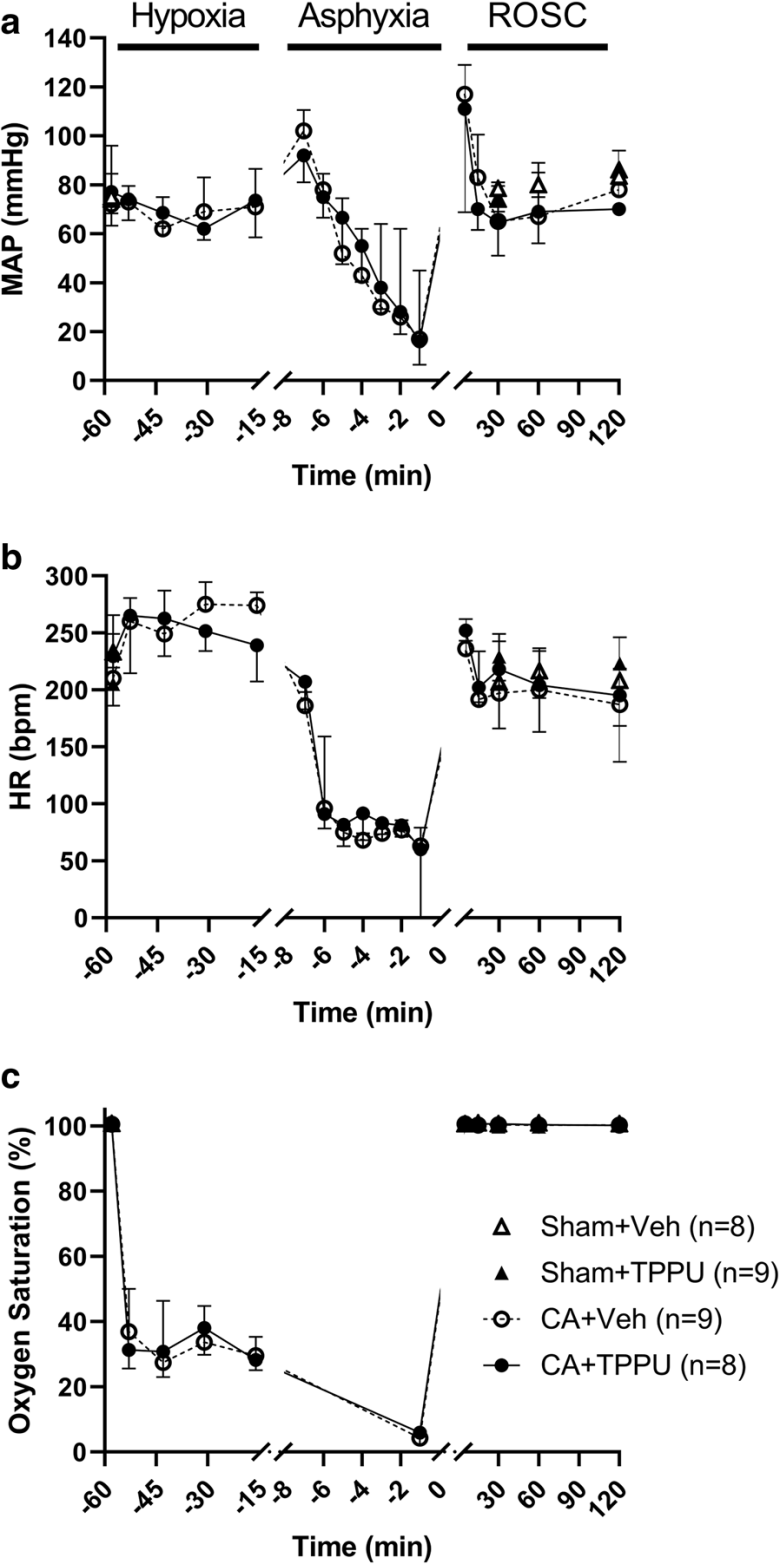
Mean arterial pressure (MAP; **a**), heart rate (HR; **b**), and oxygen saturation (**c**) during 45 min of hypoxia, 7 or 8 min of asphyxia, and 120 min of recovery. Time zero represents the return of spontaneous circulation (ROSC). Data are displayed as median and interquartile range. CA, cardiac arrest; Veh, vehicle

Baseline characteristics for the 4-day survivors in each of the 4 groups are shown in Table 2 (n = 8–9/group). Physiologic parameters were normal for swine. The groups exhibited no differences except for a significantly lower pH and greater base excess in the Sham + TPPU group, although the pH was still within normal clinical limits (7.35-7.45). At 42 min of hypoxia, the oxygen saturation was similar in the CA + vehicle and CA + TPPU groups (29.5% [26.3, 35.3] versus 28.3% [25.1, 37.2], *p *= 0.815). During asphyxia, piglets developed the expected decline in mean arterial pressure (MAP), heart rate, and oxygen saturation (Fig. 1). Median MAP at 1 min before the end of asphyxia was 17 mmHg [11, 45] in the CA + vehicle group and 16 mmHg [7, 48] in the CA + TPPU group (*p *= 0.869). Median duration of resuscitation was 30 s [20, 83] in the CA + vehicle group and 35 s [20, 133] in the CA + TPPU group (*p *= 0.869). Piglets in the CA + vehicle group received a median of 1 (0, 1.5) dose of epinephrine versus 0.5 (0, 1.8) dose in the CA + TPPU group (*p *= 0.997). In the CA + vehicle group, 25% of piglets required defibrillation for ventricular fibrillation versus 50% in the CA + TPPU group (*p *= 0.335). There was no difference in MAP between animals that received vehicle and those that received TPPU at 30 min after administration (60 min after return of spontaneous circulation [ROSC], 76 mmHg [66, 89] versus 73 mmHg [62, 85], respectively, *p *= 0.570).

**Table 2:** Baseline characteristics of the 4 experimental groups

Parameter	Sham + Veh (n = 8)	Sham + TPPU (n = 9)	CA + Veh (n = 9)	CA + TPPU (n = 8)	*p*
Weight (kg)	3.75 [3.54, 4.09]	3.44 [3.26, 3.88]	3.52 [3.44, 3.82]	3.53 [3.24, 3.68]	0.297
ETCO_2_ (torr)	46 [42, 49]	47 [44, 54]	48 [46, 50]	44 [36, 48]	0.293
Temperature (°C)	38.2 [38.0, 38.5]	38.5 [37.9, 38.6]	38.8 [38.1, 39.3]	38.7 [37.8, 38.9]	0.571
MAP (mmHg)	75 [64, 85]	74 [71, 94]	72 [68, 83]	75 [70, 84]	0.869
HR (bpm)	233 [187, 268]	205 [190, 217]	207 [185, 261]	228 [216, 246]	0.944
Arterial pH	7.41 [7.38, 7.45]	7.36 [7.34, 7.38]^a^	7.39 [7.37, 7.42]	7.43 [7.41, 7.47]	0.001
PaCO_2_ (torr)	33 [32, 36]	36 [33, 39]	38 [37, 39]	35 [32, 37]	0.104
PaO_2_ (torr)	169 [144, 172]	159 [151, 167]	161 [152, 171]	153 [134, 217]	0.987
BE (mmol/L)	− 2.6 [− 4.7, 0.5]	− 5.1 [− 5.4, − 2.8]	− 1.7 [− 3.0, − 0.5]	0.2 [− 3.3, 2.5]	0.047
Hb (g/dL)	11.0 [10.6, 11.2]	10.5 [9.3, 11.4]	10.7 [10.1, 11.0]	11.0 [10.8, 11.5]	0.721
Na (mmol/L)	137 [134, 140]	140 [135, 141]	140 [138, 141]	139 [135, 141]	0.766
Glu (mg/dL)	103 [101, 123]	142 [125, 166]	160 [127, 199]	121 [83, 135]	0.077

All data are presented as median and interquartile range. Data were analyzed by Kruskal–Wallis test with post hoc Dunn’s multiple comparisons test

BE, base excess; bpm, beats per min; CA, cardiac arrest; ETCO_2_, end-tidal CO_2_; Glu, glucose; Hb, hemoglobin; HR, heart rate; MAP, mean arterial pressure; Na, sodium; PaCO_2_, arterial partial pressure of carbon dioxide; PaO_2_, arterial partial pressure of oxygen;Veh, vehicle

^a^*p *< 0.001 vs CA + TPPU

### Neuropathology outcomes in putamen and motor cortex

**Fig. 2:**
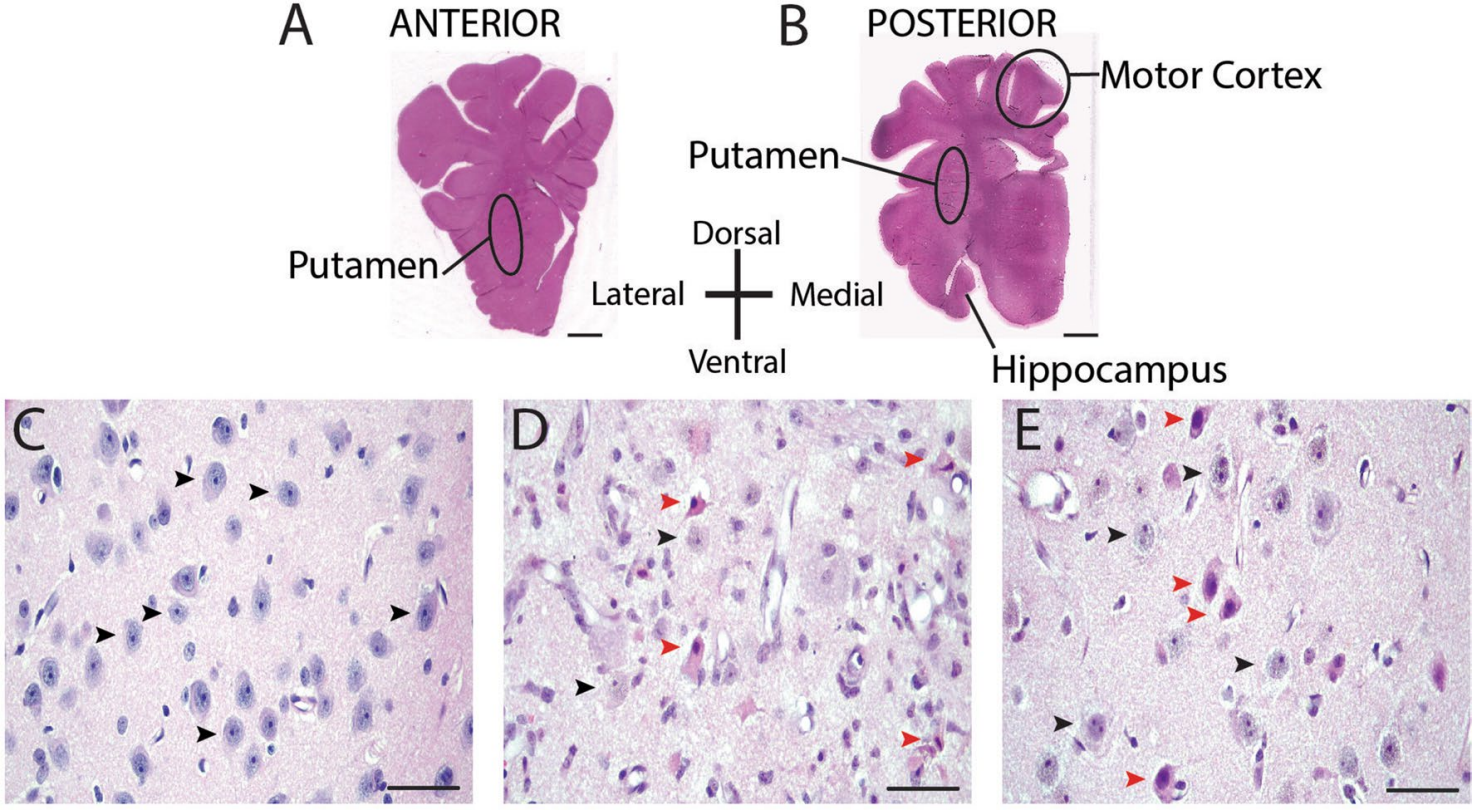
Representative images of brain sections from piglets that underwent sham surgery or cardiac arrest (CA) injury. **a**, **b** Macrophotographs show H&E-stained sections of anterior and posterior putamen and motor cortex where neurons were counted in delineated areas. Brain section orientation is indicated. Scale bar is 5 mm. **c** H&E-stained putamen in a sham-operated pig that received vehicle treatment. Black arrowheads denote morphologically normal neurons. **d** H&E-stained putamen in a CA + vehicle piglet shows morphologically normal neurons (black arrowheads) and typical ischemic-necrotic morphology denoted by red arrowheads. The ischemic-necrotic neurons have a shrunken, angular cell body; eosinophilic cytoplasm; and a condensed, eccentrically placed nucleus. **e** H&E-stained putamen in a sham-operated piglet that received TPPU. Black arrowheads denote morphologically normal neurons and red arrowheads denote injured neurons characterized by a round, condensed, and eccentrically placed nucleus, presence of nucleolus, and eosinophlic cytoplasm. The photos in c, d, and e were taken at ×400 magnification and the scale bar is 50 μm

We analyzed anterior putamen at the septal anatomic level and posterior putamen and cerebral cortex at the hippocampal anatomic level in one hemisphere with hematoxylin and eosin (H&E) stain (Fig. 2a, b). The motor cortex was the cortical area analyzed. This gyrus in pig is the origin site of the corticospinal tract [19]. These regions are reliably damaged in piglet CA [20], and neurodegeneration in these regions, notably the striatum, is sensitive to some experimental therapies administered acutely and short term [21–23]. We counted microscopically normal, ischemic, and injured neurons. This classification scheme has been defined and used before [43], and examples of each classification are shown in Fig. 2c–e. Briefly, normal neurons (Fig. 2c, e, black arrowheads) had a round cell body (generally 8-10 μm in diameter); an open nucleus that contained visible chromatin strands within a diffuse nucleoplasmic matrix with a nucleolus; uninterrupted nuclear and cellular membranes; and a normal thin rim of cytoplasm [24]. The ischemic-necrotic cytopathology [25] (Fig. 2d, red arrowheads) had a shrunken, acutely angular cell body with homogeneous glassy, eosinophilic cytoplasm that contained microvacuoles and a hematoxylin-stained attritional and angular nucleus with dark speckling of the nucleoplasmic matrix and no nucleolus [20]. Injured neurons (Fig. 2e, red arrowheads) had apparently intact cellular and nuclear membranes, condensed and round nucleus often still with a nucleolus, and hypereosinophilic cytoplasm with multiple vacuoles [26].

Counts of ischemic-necrotic neurons in the three anatomic areas are reported first because this information indicates active neurodegeneration (Table 3). In anterior putamen, piglets that underwent CA with vehicle treatment had significantly more ischemic-necrotic neurons/mm^2^ than did vehicle-treated shams (43 [37, 93] versus 3 [1, 7] neurons/mm^2^, *p *= 0.026). The number of ischemic-necrotic neurons after CA did not differ between the TPPU-treated group (68 [24,107] neurons/mm^2^) and the vehicle-treated group (*p *> 0.999). The number of ischemic-necrotic neurons/mm^2^ in posterior putamen was significantly greater in the CA + vehicle group than in the Sham + vehicle group (43 [8, 80] versus 1 [0, 5] neurons/mm^2^, *p *= 0.013) or the Sham + TPPU group (2 [0, 5], *p *= 0.028). However, among piglets that underwent CA, the number did not differ significantly, regardless of whether piglets were treated with vehicle or TPPU (43 [8, 80] versus 20 [3, 65], *p *> 0.999). In motor cortex, no difference in the number of ischemic necrotic neurons was apparent among the four groups (*p *= 0.053).

**Table 3:** Ischemic neurons per mm^2^ in the 4 experimental groups at 4 d recovery

Anatomic area	Sham + Veh (n = 8)	Sham + TPPU (n = 9)	CA + Veh (n = 9)	CA + TPPU (n = 8)	*p*
Anterior putamen	3 [1, 7]	1 [0, 3]	43 [37, 93]^a^	68 [24, 107]^b^	< 0.001
Posterior putamen	1 [0, 5]	2 [0, 5]	43 [8, 80]^c^	20 [3, 65]	0.003
Motor cortex	1 [1, 2]	4 [1, 7]	8 [4, 14]	6 [3, 13]	0.053

Data are presented as median and interquartile range. Data were analyzed by Kruskal–Wallis test with post hoc Dunn’s multiple comparisons test

CA, cardiac arrest; Veh, vehicle

^a^*p *= 0.026 vs Sham + Veh and *p *< 0.001 vs Sham + TPPU

^b^*p *= 0.016 vs Sham + Veh and *p *< 0.001 vs Sham + TPPU

^c^*p *= 0.013 vs Sham + Veh and *p *= 0.028 vs Sham + TPPU

**Fig. 3:**
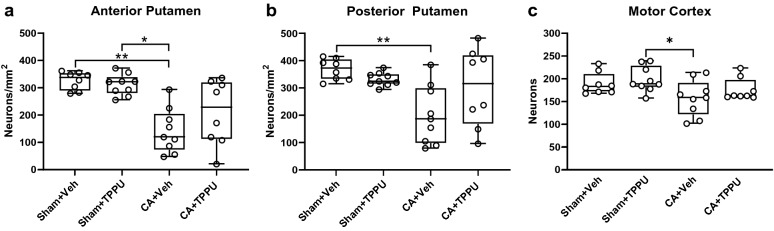
Neuron counts per mm^2^ in **a** anterior putamen, **b** posterior putamen, and **c** motor cortex in the 4 experimental groups. *p < 0.05, **p < 0.01 versus the indicated group. Data are displayed as median and interquartile range with 5-95% whiskers. CA, cardiac arrest; Veh, vehicle

Figure 3 shows counts for the remaining non-ischemic neuronal cell densities in putamen of the four experimental groups at 4 days of recovery. In anterior putamen (Fig. 3a), the number of neurons/mm^2^ was significantly different among the four groups (*p *= 0.001). In the Sham + vehicle and Sham + TPPU groups, the number of neurons/mm^2^ was similar (337 [288, 354] and 322 [279, 340] neurons/mm^2^, respectively; *p *> 0.999). After CA, vehicle-treated piglets had significantly fewer neurons (120 [72, 205] neurons/mm^2^) than did vehicle-treated shams (*p *= 0.003) or TPPU-treated shams (*p *= 0.015). There was no difference in the number of neurons/mm^2^ between the CA + vehicle and the CA + TPPU groups (120 [72, 205] versus 228 [111, 320] neurons/mm^2^, *p *> 0.999). The number of neurons/mm^2^ in the CA + TPPU group was also similar to that in the Sham + vehicle and Sham + TPPU groups (*p *= 0.191 and *p *= 0.620, respectively).

In posterior putamen (Fig. 3b), the number of neurons/mm^2^ was significantly different among the four groups (*p *= 0.010). The number of neurons/mm^2^ did not differ significantly between shams that received vehicle and those that received TPPU (373 [332, 405] versus 324 [314, 351] neurons/mm^2^, *p *> 0.999). Piglets that underwent CA and vehicle treatment had significantly fewer neurons/mm^2^ than did vehicle-treated shams (187 [97, 300] versus 373 [332, 405] neurons/mm^2^, *p *= 0.006). Treatment with TPPU after CA did not improve the number of neurons/mm^2^ above that observed in the CA + vehicle group (316 [168, 420] versus 187 [97, 300], *p *= 0.184). Neuronal counts also did not differ significantly between the CA + TPPU group and the Sham + vehicle (*p *> 0.999) or Sham + TPPU group (*p *= 0.413).

In motor cortex (Fig. 3c), the number of neurons was different among the four groups (*p *= 0.038). Again, we found no difference in the number of neurons in layers II-VI between the Sham + vehicle and Sham + TPPU groups (183 [171, 211] versus 189 [182, 229] neurons, respectively, *p *> 0.999). Piglets that underwent CA and vehicle treatment had fewer neurons than did shams that received TPPU treatment (159 [121,191] versus 189 [182,229] neurons, *p *= 0.048). TPPU treatment after CA did not improve the number of neurons above that observed in the CA + vehicle group (163 [161, 198] versus 159 [121, 191] neurons, respectively, *p *> 0.999).

In sham piglets treated with TPPU, 20% of neurons in both anterior and posterior putamen had abnormal morphology with cell body attrition, nuclear condensation with intact nucleolus, and hypereosinophilic cytoplasm (Fig. 2e, red arrowheads). This pathology was observed in < 5% of neurons of the motor cortex in all four treatment groups.

### Neurologic deficits

**Fig. 4:**
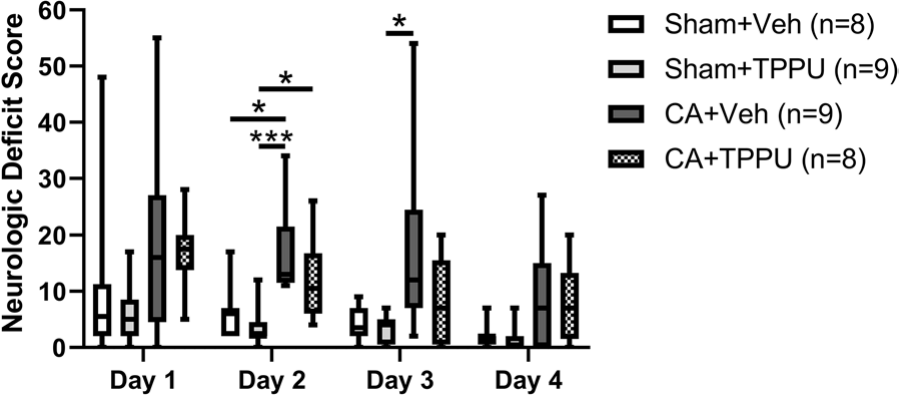
Neurologic deficit scores in the 4 experimental groups on days 1–4 after cardiac arrest (CA) injury. The neurologic deficit score assesses consciousness, motor and sensory function, spatial orientation, excitability, and presence of seizures on a 154-point scale. Higher scores are indicative of greater injury. *p < 0.05, ***p < 0.001 versus indicated group. Veh, vehicle

The neurologic deficit score (NDS) was measured in all 34 piglets on days 1 and 2 after injury or sham surgery. Data were not collected from one pig in the Sham + TPPU group on day 3 or from two CA + TPPU piglets, two CA + vehicle piglets, and one Sham + TPPU pig on day 4 due to limited lab personnel. The NDS after CA was most severe at day 1 and improved in all groups over the 4-day recovery period (Fig. 4). After 2 days of recovery, the CA + vehicle group had a significantly worse NDS than either the Sham + vehicle group (13 [12, 22] versus 6 [2, 7], *p *= 0.035) or the Sham + TPPU group (2 [2, 5], *p *< 0.001). NDS was also worse in the CA + TPPU than in the Sham + TPPU group at this time (11, [6 17] versus 2 [2, 5], *p *= 0.035). After 3 days of recovery, NDS remained worse in the CA + vehicle group than in the Sham + TPPU group (12 [7, 25] versus 4 [1, 5], *p *= 0.021). NDS did not differ between piglets that received post-CA vehicle and those that received post-CA TPPU at any time point. All groups had similar weight gain at 4 days of recovery (*p *= 0.070). Two piglets in the CA + vehicle group had clinical seizures on days 1 and 2 of recovery and were treated with intravenous phenobarbital. No other piglets had observed clinical seizures.

### Proteins markers of ER stress

**Fig. 5:**
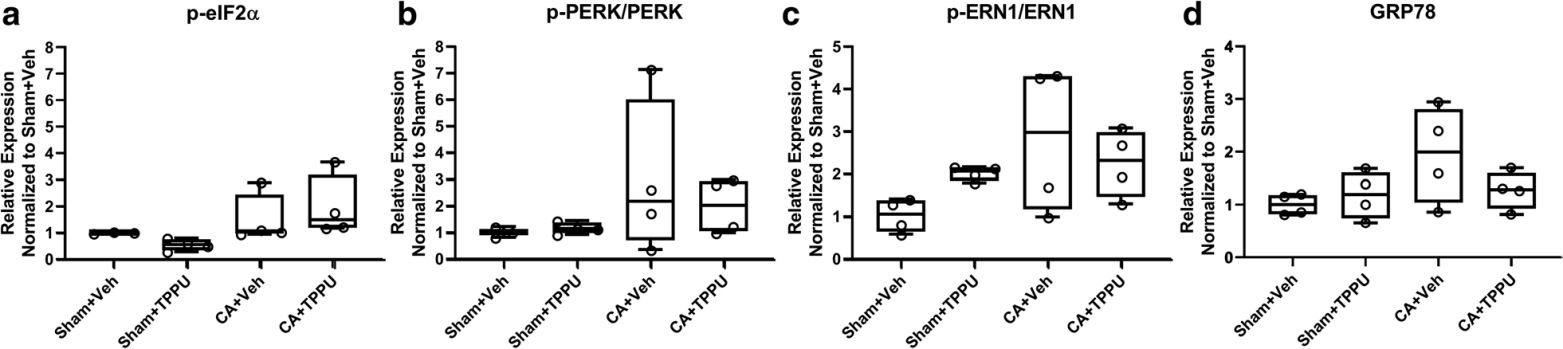
Protein markers of endoplasmic reticulum stress. Western blotting showed similar expression of p-eIF2α (**a**, *p *= 0.167), p-PERK/PERK (**b**, *p *= 0.167), p-ERN1/ERN1 (**c**, *p *= 0.167), and GRP78 (**d**, *p *= 0.375) in putamen at 3 h of recovery. Data were normalized to the Sham + vehicle group and analyses conducted by Friedman test with data blocked by gel. CA, cardiac arrest; Veh, vehicle

A putative mechanism for TPPU neuroprotection is modulation of ER stress [18]. We therefore interrogated whether TPPU might provide biochemical protection not reflected by the neuropathological analysis. On western blotting we found no differences in the expression of phosphorylated eIF2α (*p *= 0.167), the ratio of phosphorylated PERK/PERK (*p *= 0.167), the ratio of phosphorylated ERN1/ERN1 (*p *= 0.167), or GRP78 (*p *= 0.375) among the four experimental groups at 3 h of recovery (Fig. 5).

## Discussion

We show that intravenous treatment with an sEH inhibitor at 30 min and 24 h after asphyxic CA does not protect neurons in putamen or motor cortex at 4 days of recovery in this swine model. Furthermore, treatment with TPPU does not improve daily neurologic deficit scores above those observed in piglets administered vehicle after CA. Despite efficacy in rodent models of global [16, 17] and focal hypoxic brain injury [15, 27, 28], short-term therapy with an sEH inhibitor is not neuroprotective after CA in this pediatric swine model.

There are several considerations for the lack of neuroprotection in this study. We used a TPPU dose of 1 mg/kg based on our own and others’ experiences with a rodent model of focal ischemic brain injury [15, 28]. To our knowledge, TPPU has not been used in swine injury models, and the pharmacokinetics may differ from that of rodents and cynomolgus monkeys, in which the half-life is approximately 10 h [29] and 30 h [30], respectively. Although this dose is expected to achieve blood concentrations of TPPU [31] well above the half maximal inhibitory concentration of sEH in humans and non-human primates [30] and is reported to effectively penetrate the uninjured blood–brain barrier in rodents [32], it is possible that swine require different TPPU doses or dose regimens. We did not have a brain bioavailability assessment for TPPU. Moreover, the potency of different sEH inhibitors for sEH can differ markedly among species [30, 33]. Furthermore, discrepant outcomes have been reported with both higher- and lower-than-optimal TPPU doses in studies examining dose escalation [28, 34–36].

Another consideration is that we may have administered TPPU too late after the injury to achieve protection. However, other drugs have been shown to be neuroprotective in a similar piglet model with comparable therapeutic timing [22, 23]. We chose to administer the first dose of TPPU at 30 min after ROSC to mimic clinical delays that occur when an unstable patient is in the intensive care setting, and based on demonstrated neuroprotection with this dosing regimen after CA in mice [17]. However, it is possible that earlier administration after CA is necessary for neuroprotection, especially since molecular pathways of cell death are activated within 15 min of injury in this swine model [37]. One study in mice demonstrated neuroprotection when an sEH inhibitor was administered within 5 min of ROSC [16], and other stroke models have suggested a greater benefit when sEH inhibitor is administered earlier, such as before injury [13, 27, 38], at the onset of ischemia [28], or at the time of reperfusion [15]. Another explanation for the apparent species differences in the neuroprotective efficacy of TPPU could be that neuronal responses and cell death mechanisms are differential and nuanced in rodents and pigs [39] or depend upon developmental stage. In an adult mouse CA model, sEH inhibitors provided benefit to the hippocampus but not to the caudate-putamen [16, 17]. In immature piglets, however, the CA1 hippocampus undergoes very little delayed neurodegeneration after CA [40]. Cell death in piglet putamen is rapid and necrotic; suppression of the subsequent inflammatory response by an sEH inhibitor may be inconsequential to the rapidly evolving fate of striatal neurons. Furthermore, the ischemia elicited by brief cardiac arrest and the resultant selective neuronal necrosis cannot be equated with the prolonged ischemia of middle cerebral artery occlusion, where a robust neuroinflammatory microenvironment is ignited by the pancellular neurodegeneration.

It is also possible that the 24-h duration of TPPU therapy in this study was insufficient. Other studies have demonstrated neuroprotection with daily dosing of sEH inhibitors for 3 days after MCAO [28, 41]. Moreover, we have previously shown that neuronal cell death peaks at 24 h in striatum [42] but is progressive over 4 days [20]. In motor cortex, peak injury is delayed [20] and might be exacerbated by development of seizures on days 1–2 of recovery [40, 43]. Therefore, additional studies examining extension of TPPU therapy through this critical window of brain injury progression are necessary.

Inactivation of sEH could have deleterious effects on injury mechanisms after CA, or sEH activity in other organs may be necessary for recovery. In a mouse CA model, Hutchens et al. [44] showed that mortality was significantly greater in sEH knockout mice (100% at 24 h) than in wild-type mice. Although survival after CA was similar between the vehicle and TPPU-treated groups in our study, this observation raises the possibility that sEH is important for recovery of the heart, and potentially other organs, after whole-body ischemia. Hutchens et al. [44] postulated that the poor survival was due to the EETs having a potent vasodilatory effect that limited myocardial perfusion. However, we observed no difference in MAP between the vehicle- and TPPU-treated groups at 1 h of recovery.

In keeping with the idea that TPPU might have deleterious actions, our data also suggest that it may have unintended consequences in uninjured brain. In basal ganglia, a small but significant proportion of neurons appeared morphologically abnormal in the Sham + TPPU group. Because a favorable safety profile has been reported in a phase 1 trial of an sEH inhibitor [45], the fate of these abnormal neurons should be studied in depth before TPPU can be further studied in immature or injured brain, and before sEH inhibitors in general are advanced clinically.

We initially hypothesized that TPPU would afford neuroprotection and that the mechanism might be through attenuation of ER stress. We did not observe neuroprotection neuropathologically but hypothesized that TPPU still could exert biochemical actions not registered by histology. We observed no difference in key ER stress protein levels between the Sham + vehicle and the CA + vehicle groups at 3 h of recovery. Additionally, TPPU did not change levels of key ER stress proteins in striatum. In neonatal mouse hypoxic brain injury [46], acute ER stress is evident biochemically at 3 h post-injury and appears persistent. In piglet, acute ER stress has been directly visualized in putamen neurons by electron microscopy and biochemically by release of ER-sequestered proteins at 3-6 h [47]. It is possible that the 3 h time point was too early to sufficiently capture ER stress after CA in this model. More work is needed to determine the timing and selectivity of the ER stress response after hypoxia–ischemia in piglet.

### Limitations

Our results should be considered in the context of several limitations. First, we did not examine whether TPPU effectively inhibited brain sEH activity in this swine model. Pharmacologic and metabolomic studies of TPPU in swine are needed. Second, variability in the number of neurons after CA was unexpectedly high in both the vehicle- and TPPU-treated cohorts. It is therefore possible that the study was underpowered with an n of 8–9 piglets per group to detect a difference between groups. The variable degree of injury observed might be explained by subclinical seizures. The incidence of clinical seizures was low in this cohort (observed in only 10% of piglets) but it is possible that some piglets suffered from confounding subclinical seizures that contributed to worse pathologic injury. Finally, although the NDS revealed differences between the CA and sham groups, the NDS is a coarse measure of behavior and motor and sensory function. Given reports of more subtle neurocognitive deficits in survivors of CA, additional paradigms of neurocognitive testing in swine are necessary.

## Conclusions

We conclude that treatment with the sEH inhibitor TPPU after CA does not protect neurons in basal ganglia or motor cortex and does not improve neurologic deficits in this pediatric swine cohort. Additional study is needed to establish whether TPPU is neuroprotective after CA in swine.

## Methods

### Study design

We performed a prospective, randomized controlled study to compare the effects of TPPU and vehicle treatment on neurologic outcomes at 4 days of recovery in a swine model of pediatric CA.

### Animal preparation

All procedures were approved by the Animal Care and Use Committee at Johns Hopkins University and complied with the National Institute of Health guidelines for the care of animals in research [48]. Male, Yorkshire swine weighing 3–4 kg were housed in groups of three and fasted overnight prior to the experiment. We previously published a similar hypoxic-asphyxic cardiac arrest injury protocol [24, 26, 49]. Briefly, piglets were anesthetized via nose cone with 5% isoflurane in a 50%/50% nitrous oxide/oxygen mixture. After intubation, mechanical ventilation was initiated to maintain normocapnia and normoxia. The inhaled oxygen was decreased to 30% in a 70%/30% nitrous oxide/oxygen mixture, and the isoflurane was decreased to 2% during the surgical procedures. Under sterile conditions, the external jugular vein and femoral artery were exposed and cannulated for intravenous (IV) administration of medications and continuous hemodynamic monitoring, respectively. The isoflurane was discontinued after the surgical procedures, which take an average of 30 min, and intermittent IV doses of fentanyl (10 µg/kg) and vecuronium (1 mg/kg) were provided for animal comfort.

### Global hypoxic injury and cardiac arrest

Piglets were randomized to sham surgery or global hypoxia–ischemia injury by use of a random number generator to minimize potential confounders including litter and procedural table (we use three different surgical/experimental stations simultaneously). We induced whole-body hypoxia by decreasing the inhaled oxygen to 10% for 45 min to achieve a goal oxyhemoglobin saturation of 30%. Inhaled oxygen was decreased by titrating nitrogen gas into the ventilation circuit. The piglets then received room air for 5 min. We have found that this brief reoxygenation period is required for cardiac resuscitation. We then occluded the endotracheal tube to produce an asphyxial CA. The first cohort of animals underwent 8 min of asphyxia, but survival was poor. Therefore, we amended the protocol to 7 min, which improved survival. Most piglets suffered a bradycardic CA. Piglets were then resuscitated with 100% oxygen, manual chest compressions, and IV epinephrine (100 μg/kg) when clinically indicated. Defibrillation at 30 joules (10 J/kg) was also provided when ventricular fibrillation occurred. Piglets that did not achieve ROSC within 3 min were excluded from the experiment in an effort to standardize the injury. After resuscitation, the inhaled oxygen was decreased to 30% for the remainder of the experiment. Sodium bicarbonate was administered to correct metabolic acidosis as necessary. Piglets were maintained at 38–39.5 °C (normothermia for swine) with heating blankets during recovery. Sham-operated piglets received the same external jugular and femoral cannulae, duration of anesthesia, and 30% inhaled oxygen. After 3 h of recovery, piglets were extubated and returned to their cages. During the 4-day recovery, an investigator blinded to group quantified neurologic deficits with a scoring system (neurologic deficit score, NDS) that evaluates consciousness; brainstem, sensory, and motor function; behavior; spatial orientation; excitability; and prescence of seizures on a 154-point scale [50].

### TPPU and vehicle administration

Piglets received either the sEH inhibitor 1-trifluoromethoxyphenyl-3-(1-propionylpiperidin-4-yl) urea (TPPU) or vehicle treatment, producing four experimental groups: (1) Sham + vehicle, (2) Sham + TPPU, (3) CA + vehicle, and (4) CA + TPPU. TPPU was generously provided by the laboratory of Dr. Bruce Hammock (UC Davis, USA) and stored at 20 °C. TPPU was dissolved in poly(ethylene glycol) 400 (Sigma Aldrich, Darmstadt, Germany) and diluted to a 10% poly(ethylene glycol) solution with sterile water. TPPU or vehicle (10 mL) was administered intravenously at 30 min after ROSC and again at 24 h of recovery for a total of 2 doses. This dose and dosing interval were chosen based on prior work that showed neuroprotection after unilateral hypoxic injury in rodents [15, 28] and on work that demonstrated neuroprotection after cardiac arrest in mice [17].

### Histology

At 4 days of recovery or time equivalent in shams, piglets were deeply anesthetized and euthanized with IV injections of pentobarbital 50 mg/kg plus phenytoin 6.4 mg/kg and then transcardially perfused with cold phosphate-buffered saline followed by ~ 4 L of ice-cold 4% paraformaldehyde for brain fixation. The head was stored overnight at 4 °C for in situ fixation. Afterwards, we carefully removed the brain from the skull, cut it midsagittally, and immersed the right hemisphere in 4% paraformaldehyde for postfixation. The hemisphere was cut into 1-cm slabs that were embedded in paraffin and cut into 10-μm coronal sections.

One investigator (CEO), who was blinded to the treatment group, counted normal, ischemic, and injured neurons in each area by light microscopy at 400× magnification. In putamen, a total of 8–10 nonoverlapping fields of 0.225 mm^2^ per piglet were counted. Cells were also counted in cortical layers 2–6 of the motor gyrus at 400× magnification in 6 nonoverlapping fields. These cortical layers were reliably identified and the motor gyrus was identified as the most medial gyrus to reach the dura [51]. Counter reliability was screened for accuracy by a second investigator (LJM). Representative images of normal, ischemic, and injured neurons are shown in Fig. 2c–e. Since the fate of the injured neurons was unknown, normal and injured neuron counts were combined for a total neuron count.

### Immunoblotting

We prepared a second cohort of piglets for biochemical experiments to evaluate TPPU’s mechanism of action. Piglets were sacrificed at 3 h after ROSC or an equivalent time after sham procedure. We chose the 3 h time point because nascent damage in ER of putamen is seen ultrastructurally by cisternal swelling and ribosomal undocking, and biochemically by release of KDEL-motif proteins into the soluble compartment at 3–6 h of survival [47]. Piglets were then deeply anesthetized, euthanized, and transcardially perfused with cold phosphate-buffered saline. Brain tissue was immediately harvested. Putamen was dissected from fresh brain slabs on dry ice and stored at − 80 °C.

Brain samples were homogenized in ice-cold RIPA buffer (Cell Signaling Technology, Danvers, MA, USA), protease inhibitor cocktail (Invitrogen, Grand Island, NY, USA), and phosphatase inhibitor (Roche Applied Science, Mannheim, Germany) at a ratio of 1 mL per 0.1 g putamen tissue. After the homogenates were centrifuged at 4 °C, protein concentrations in the supernatant were measured with the Pierce BCA protein assay kit (Thermo Scientific, Rockford, IL, USA). Samples were treated with loading buffer, boiled for 5 min, separated by sodium dodecyl sulfate–polyacrylamide gel electrophoresis on 4%–12% Tris–glycine gels, and transferred to nitrocellulose membranes. Each gel contained a pig putamen homogenate sample from each treatment group. After transfer, the membranes were stained with Ponceau S (Sigma Life Science, St. Louis, MO, USA) and imaged for quantification of protein loading. After being washed, the membranes were blocked in 5% nonfat milk for 1 h at room temperature and then incubated overnight with primary antibody in 2% milk at 4 °C. The membranes were incubated in anti-mouse IgG (Jackson ImmunoResearch, West Grove, PA, USA) or anti-rabbit IgG (GE Healthcare, Nottingham, UK) diluted 1:3000 in 2% milk for 2 h at room temperature. The membranes were imaged with enhanced chemiluminescence (Bio-Rad, Hercules, CA, USA) and iBrightCL1000 Imaging System (Invitrogen). Immunoreactive band densities were analyzed with MyImageAnalysis v2.0 (Thermo Fisher Scientific, Waltham, MA, USA). The densities were normalized to Ponceau to account for protein loading variability and then to the Sham + vehicle group as the control on each gel. Sizes of the proteins of interest were determined by using a molecular weight reference ladder (Precision Plus Protein Standards, BioRad). The following immunoreactive bands were measured: phosphorylated eukaryotic translation initiation factor 2α (p-eIF2α, 36 kD, Abcam), protein kinase-like endoplasmic reticulum kinase (PERK, 140 kD, Cell Signaling Technology), phosphorylated PERK (p-PERK, 170 kD, Biolegend, San Diego, CA, USA), endoplasmic reticulum-to-nucleus signaling 1 (ERN1, 110 kD, Novus Biologicals, Centennial, CO), phosphorylated ERN1 (p-ERN1, 110 kD, Novus Biologicals), and glucose-regulated protein (GRP78, 78 kD, Santa Cruz Biotechnology, Dallas, TX, USA).

### Sample size calculations

We had no a priori knowledge of the magnitude of change in number of viable neurons in the basal ganglia with TPPU treatment after cardiac arrest in this model. Therefore, we made a sample size estimate after the first 3 animals underwent CA + vehicle treatment and another 3 animals underwent Sham + vehicle treatment. The mean number of viable neurons in putamen in the 3 CA + vehicle animals was 70.7 ± 39.2 neurons/mm^2^, which represented a loss of 80% of neurons compared to the 3 Sham + vehicle animals. We estimated a sample size based on a 25% improvement in the number of viable neurons with TPPU treatment. With an alpha of 0.05 and a power of 80%, we calculated that we would need 7 animals/group. We increased our sample size to 8-9 piglets/group to account for error in our estimations of variability.

### Statistical analysis

Analyses were conducted and graphs generated with Stata (v15.1, StataCorp, College Station, TX, USA) and GraphPad (v8.4.2, GraphPad Software, La Jolla, CA, USA). Survival was analyzed with Fishers exact test. Physiologic data of the two CA groups were compared by Mann–Whitney U test. Pathology data are graphed as box plots with interquartile ranges (IQR) and 5-95^th^ percentile whiskers. To evaluate the effects of TPPU on total and ischemic neuron counts, we used Kruskal–Wallis one-way analysis of variance by ranks. Post-hoc multiple comparisons were conducted with Dunn’s multiple comparisons test. *P* values were adjusted for multiple comparisons and *p *< 0.05 was considered significant. Immunoblot densities were normalized to (divided by) that of Sham + vehicle piglets and then analyzed by Friedman repeated measures analysis of ranks with data blocked by gel.

## Data Availability

The datasets used and/or analyzed during the current study are available from the corresponding author on reasonable request.

## Abbreviations

CA: Cardiac arrest
EETs: Epoxyeicosatrienoic acids
ER: Endoplasmic reticulum
ERN1: Endoplasmic reticulum-to-nucleus signaling 1
GRP78: Glucose-regulated protein
H&E: Hematoxylin and eosin
IQR: Interquartile range
IV: Intravenous
NDS: Neurologic deficit score
p-ERN1: Phosphorylated endoplasmic reticulum-to-nucleus signaling 1
p-eIF2α: Phosphorylated eukaryotic translation initiation factor 2α
p-PERK: Phosphorylated protein kinase-like endoplasmic reticulum kinase
PERK: Protein kinase-like endoplasmic reticulum kinase
sEH: Soluble epoxide hydrolase
TPPU: 1-Trifluoromethoxyphenyl-3-(1-propionylpiperidin-4-yl) urea
